# Entering Higher Professional Education: Unveiling First-Year Students’ Key Academic Experiences and Their Occurrence Over Time

**DOI:** 10.3389/fpsyg.2021.577388

**Published:** 2021-02-24

**Authors:** Jonas Willems, Liesje Coertjens, Vincent Donche

**Affiliations:** ^1^Department of Training and Education Sciences, Faculty of Social Sciences, University of Antwerp, Antwerp, Belgium; ^2^Psychological Sciences Research Institute, Université Catholique de Louvain, Louvain-la-Neuve, Belgium

**Keywords:** transition to higher education, professional higher education, academic adjustment, academic integration, critical incidents, first-year experience

## Abstract

To date, little understanding exists of how first-year students in professionally oriented higher-education (HE) programs (i.e., those that provide vocational education to prepare students for a particular occupation) experience their academic transition process. In the present study, we first argued how the constructs of academic adjustment and academic integration can provide complementary perspectives on the academic transition of first-year students in (professional) HE. Next, we examined what first-year students in professional HE contexts perceive to be the most important experiences associated with their academic transition process in the first semester of their first year of higher education (FYHE). To this end, we adopted the fundamentals of the critical incident technique and asked 104 students in a Flemish (Dutch-speaking part of Belgium) university college (which offers professional HE programs, such as nursing) to complete “reflective logs” with open questions at the start of the second semester of their FYHE, wherein they reflected on three critical academic experiences during their first semester. An inductive, cross-case content analysis of the collected narratives showed that students reported on nine themes of academic experiences, which relate to five adjustment themes (dealing with the organization of the study program, organizing study work, committing to the study, following class and taking notes, and processing learning content outside class) and four integration themes (feeling competent, feeling stressed, feeling prepared, and feeling supported). Further analyses showed that although some of the nine themes of academic experiences appear to be more important at different times in the first semester, they all seem to be meaningful throughout the whole semester.

## Introduction

In the last decades, the transition of students from high school to their first year of higher education (FYHE) has received extensive research attention, as it is generally recognized that the first-year student experience impacts important student outcomes, such as academic success and well-being (Bowman, 2010; Gale and Parker, 2014). This transition for many students is challenging, not in the least because students are confronted with an array of new academic requirements to which they must adapt (Credé and Niehorster, 2012). Previous research has shown that a successful transition necessitates effectively navigating academic challenges, such as managing a high workload or developing new academic skills (e.g., Trautwein and Bosse, 2017).

In this light, an increasing number of higher-education (HE) institutions have developed guidance and support initiatives to facilitate the first-year academic transition and enhance subsequent student outcomes (Coertjens et al., 2017; Harackiewicz and Priniski, 2018). Such interventions focus on various aspects of the first-year student academic experience, such as approaches to learning and perceptions of the teaching–learning environment (Ruohoniemi et al., 2017), personal development and well-being in the learning process (e.g., fear of failure and self-doubt; du Preez and McGhie, 2015), or student engagement (Hulleman et al., 2017). For the development of such guidance and support initiatives, HE institution administrators rely on guidelines based on empirical research.

However, the body of research on the first-year student academic experience has focused on academically oriented HE contexts (universities offering theoretical and scientific education) and used predominantly quantitative research designs (e.g., Baker and Siryk, 1984; Sheehan and Iarocci, 2019; Schaeper, 2020). Up till today, there has been little discussion about the academic transition of students in professionally oriented HE contexts (offering vocational education that prepares students for a specific occupation). Nevertheless, students in this latter context doubtlessly also encounter transition problems and perhaps more so, considering the higher diversity in backgrounds of students enrolling in professional HE compared with academic HE contexts (Glorieux et al., 2014). Moreover, it is clear that institutional differences might influence the academic adjustment process of first-year students (e.g., Kember and Leung, 2005; Torenbeek et al., 2013). One should thus not assume that theoretical frameworks and instruments developed in academic contexts can naturally be embedded in professional HE institutions. This lack of research in professional HE contexts is especially surprising because a significant number of adolescents worldwide participates in professional HE (OECD, 2009). For example, in Flanders (Dutch-speaking part of Belgium), 54.4% of HE students attend a professional bachelor program (Flemish Government, 2019).

Although the exact understanding and organization of academic and professional programs might vary across countries, academic programs are generally research-focused with a clear emphasis on abstract, academic-related knowledge and its development. Professional programs, on the other hand, have an emphasis on work-based education with a strong focus on the practical application of the study, adopting a curriculum that emphasizes practical aspects and elements for the development of skills and competence and includes extended phases of practical experiences in the form of internships or work experiences (Camilleri et al., 2014; OECD, 2019). Flanders, where this study was carried out, like many other HE systems, such as Germany, the Netherlands, Finland, Denmark, and Portugal, has a dual HE system, wherein academic and professional HE programs are offered at separate institutions. Flemish professional HE is offered by university colleges, which organize programs that are generally designed for learners to acquire more tailored knowledge, skills, and competencies specific to a particular occupation. Professional bachelor programs offer direct access to the labor market and coincide with the Bologna first-cycle programs (one cycle of 3 years; The Bologna Declaration, 1999). In these vocationally oriented programs, theory and practice are combined in educational practices, such as simulations, working with real-life materials, and workplace learning settings (e.g., long-term internships, machinery to repair, assignments for translators, samples to analyze; Camilleri et al., 2014).

More vocationally oriented programs in professional HE differ from academically oriented HE programs, which are offered by universities. These latter programs provide theoretical and scientific education, entailing subject matter that is more abstract and often less practical than in professional education. Also, the teaching speed is faster and more independent learning is expected from students (van Rooij et al., 2017). Academic bachelor programs typically prepare students for a master’s program and correspond with the Bologna two-cycle programs (bachelor and master’s, encompassing a total of 4 or 5 years).

The present study sets out to empirically explore how first-year students in professionally oriented programs experience their academic transition. The conceptual grounding of this study is based on former research on the first-year students’ academic experience (albeit carried out in academic HE contexts) and more in particular research on two intertwined umbrella concepts central in the transition literature and which need further investigation.

## The First-Year Academic Experience in Academic HE Contexts

In the transition literature, two “umbrella” constructs are often adopted to describe and examine the nature of the first-year academic experience, namely academic adjustment and academic integration. Although these constructs are often used interchangeably and have been described to be strongly related (e.g., Richardson et al., 2012; Rienties et al., 2012; van Rooij et al., 2017; Fematt et al., 2019; Larose et al., 2019; Veldman et al., 2019), they stem from two different, rich research traditions, which are outlined in the following paragraphs. We will explore how both the academic adjustment and integration constructs—within their respective (quantitatively oriented) traditions—describe various aspects of the academic experience and how they overlap, and we will argue how these constructs can provide complementary perspectives on the academic transition.

### Academic Adjustment: The Student Adaptation to College Model

The academic adjustment construct can be traced back to the long-established “Student Adaptation to College” (SAC) model of Baker and Siryk (1984, 1986), which was based on a review of the literature considering adjustment to college. The model postulates that the college experience is multifaceted and comprises various “demands” that require a variety of “adjustments” or “coping responses” from the individual (Baker and Siryk, 1986, p. 32). According to Baker and Siryk, one such set of demands occurs in the academic sphere of the HE institution. It is then posited that academic adjustment is the extent to which students adapt to these academic demands of the college as reflected in four subdimensions: (1) motivation for being in college and for doing academic work, (2) their engagement with academic work, (3) the effectiveness of their studying and academic efforts (e.g., trouble with concentration), and (4) the satisfaction with the academic environment (Baker and Siryk, 1986; Credé and Niehorster, 2012).

Today, the SAC framework is still widely adopted to examine freshmen’s academic adjustment to HE (e.g., van Rooij et al., 2018; Larose et al., 2019; Sheehan and Iarocci, 2019) and has proven its utility in, for instance, predicting to some extent students’ study success (e.g., review study by Credé and Niehorster, 2012). Nevertheless, the SAC framework has also received severe criticism for its lack of a strong theoretical grounding based on the paucity of information provided by the authors thereupon (see Taylor and Pastor, 2007). Indeed, the work of Baker and Siryk (1984, 1986) does not make explicit which theories they relied on for their model. It remains unclear, for instance, from where the four abovementioned subdimensions of the academic adjustment construct originate.

Another observation is that the strong quantitative (cross-sectional) focus in the SAC research tradition has generally led to a neglection of the temporal and dynamic nature of adjustment (Roland et al., 2016; De Clercq et al., 2018). Indeed, although it was not explicitly described as such in the original works of Baker and Siryk (1984, 1986), several scholars point out that adjustment should be regarded as a process in which students attempt to cope with the demands of the new environment (e.g., Anderson et al., 2016; Coertjens et al., 2017; De Clercq et al., 2018; Larose et al., 2019). In the present study, we explicitly acknowledge this process-like character of academic adjustment.

### Academic Integration: Student Attrition Framework

The academic integration construct is conceptually rooted in Tinto’s (1975) Student Attrition Framework, which was developed deductively based on Durkheim’s (1950) theory of suicide and Van Gennep’s (1960) theory of rites of passage. Tinto’s model stipulates that a freshmen’s decision to drop out or persist in his study is fundamentally based on his perceived level of social and academic integration. Over the years, the academic integration construct has acquired a central position within the research on student retention (Braxton and Hirschy, 2005), while the construct has also been adopted in other fields of transition literature, such as studies on the college experience of international students (e.g., Jean-Francois, 2019; Spencer-Oatey and Dauber, 2019).

Nevertheless, it has been pointed out that the original works of Tinto (1975, 1993) did not provide scholars with a clear definition of academic integration (Hurtado and Carter, 1997; Braxton, 2000; Brunsden et al., 2000). According to Hurtado and Carter (1997), this lack of theoretical clarity has led to a variety of operational definitions of academic integration, which reflect researchers’ various interpretations of the construct: the effort or time spent in activities; students’ perceptions, reported behaviors, and participation in specific activities; students’ satisfaction with aspects of the academic environment; objective performance criteria; or a combination of these measures (p. 326). Furthermore, together with other researchers (Davidson and Wilson, 2013; Lee et al., 2018; Tarazona and Rosenbusch, 2019), we observed that this conceptual disorientation regarding the construct of academic integration is still present in contemporary research, as recent studies consider different subfacets when operationalizing academic integration.

For instance, Schaeper (2020) conceptualized academic integration as encompassing four dimensions: (1) a structural dimension, which refers to the individual’s self-perception of meeting the standards of the HE institution; (2) a normative dimension, which concerns the individual’s identification with the normative structure of the academic system; (3) a social academic dimension, which pertains to intracurricular interactions between the individual and faculty; and (4) a motivational academic dimension, which reflects the identification of the individual with the major and their enjoyment of studying. Another recent example is the work of Ishitani (2016), who conceptualized academic integration by including how often students (1) meet with faculty, (2) meet with an academic advisor, (3) participate in study groups, and (4) talk with faculty about academic issues outside of class. Interestingly, these facets seem to partially overlap with the social academic integration dimension in the framework of Schaeper (2020). However, where Schaeper (2020) focused on the quality of the student–faculty interaction (item example: “*I feel accepted by the instructors*”), Ishitani (2016) focused on the number of such interactions (how often they occur). As a last illustration, the study by Veldman et al. (2019) takes a completely different approach to conceptualizing academic integration, comprising (1) knowing where to find and how to access university and academic support services, (2) knowing how to prepare for classes and exams, and (3) understanding and making use of the university’s academic infrastructure.

This range of multidimensional conceptualizations and operationalizations of academic integration has led scholars to label academic integration as an “umbrella” term for exploring the interrelation of the individual student with the academic system of the HE institution (Clinciu and Cazan, 2014, p. 654; Bosse et al., 2019, p. 1). Similarly, Davidson and Wilson (2013) formulated “*the terms academic and social integration have almost become … categories by which to differentiate certain predictor variables* [of student retention]” (p. 339).

### Academic Adjustment and Integration: Two Complementary Perspectives

The scant initial definitions of academic adjustment and integration, the vague theoretical groundings, and the multitude of interpretations of academic integration have obscured the overlap of the “academic integration” and “academic adjustment” constructs (see Table 1 for an overview of conceptualizations of academic adjustment and integration as described in the above-referenced studies), which are—as stated above—often used interchangeably. However, in a study that interviewed several experts in the field of transition research (Wolf-Wendel et al., 2009), Tinto clarified that the concept of academic integration should be regarded as an individual’s “*sense of belonging*” during its transition to HE and that it is a “*state of being*” based on the students’ perceptions of fit with their campus (Wolf-Wendel et al., 2009). A sense of belonging, in the transition literature can be understood as the psychological sense that one is a valued member of the HE institution community (Hausmann et al., 2007) and encompasses feelings of fitting in, acceptance, and support from a group (Strayhorn, 2012). This follows the earlier notion in the important work of Braxton (2000) that “*social and academic integration can be viewed as the psychological consequence of interactions with the institutions’ systems*” (p. 63).

**TABLE 1:** Summary of conceptualizations of academic adjustment and integration quoted in this study.

**Academic adjustment**	
Baker and Siryk, 1984, 1986	- Motivation
	- Engagement/effort
	- Effectiveness of studying and academic efforts
	- Satisfaction with academic environment
**Academic integration**	
Review by Hurtado and Carter, 1997	- Effort or time spent in activities
	- Students’ perceptions, reported behaviors, and participation in specific activities
	- Students’ satisfaction with aspects of the academic environment
	- Objective performance
	- Combination of the above
Schaeper, 2020	- Self-perceptions of meeting the standards of the HE
	- Identification with the normative structure of the academic system
	- Quality of the student–faculty interaction
	- Identification of the individual with the major and enjoyment of studying
Ishitani, 2016	- Quantity of student–faculty interaction
Veldman et al., 2019	- Knowing where to find and how to access university and academic support services
	- Knowing how to prepare for classes and exams
	- Understanding and making use of the university’s academic infrastructure

This leads us to the theoretical distinction we make between academic adjustment and academic integration. Recognizing the theoretical guidance of several scholars (e.g., Anderson et al., 2016; De Clercq et al., 2018; Larose et al., 2019), but remaining close to the initial theory of Baker and Siryk (1984), we consider academic adjustment as a process of adaptation of behavior and attitudes that may or may not enable a student to effectively meet the various academic demands encountered in the first semester of their FYHE. In accordance with Tinto (as cited by Wolf-Wendel et al., 2009) and Braxton (2000), academic integration is considered as the psychological outcome of the academic adjustment process at a certain point in time. This state of being is based on students’ perceptions of experiences within the academic sphere, reflecting their perceived fit with the new HE environment and comprising components, such as feeling supported, competent, prepared, or related to the chosen study.

In this view, academic adjustment and integration provide two complementary perspectives on the first-year academic experience: one that describes the active adaptation of behavior and attitudes as demanded by the HE environment (adjustment) and one that focuses on the psychological state of being that results from an individual’s perception of fit with the new HE environment (integration).

### Reconsidering the First-Year Academic Experience From the Students’ Perspective

Notably, conceptualizations and operationalizations of academic adjustment and integration in the abovementioned two research traditions seem to have become disconnected from the actual student experiences as reported by first-year students. Firstly, the SAC model was developed based on a literature study of SAC. It is, however, unclear how the framework offers a comprehensive picture of the first-year academic experience, as the authors do not detail where the discerned subfacets stem from or on what basis they were created. Secondly, in the last decades, scholars have given various interpretations of academic integration, focusing on different aspects of the academic experience when conceptualizing the construct (see Table 1 for illustrations). This raises the question of which experiences are *most* important according to students themselves in the process of adapting to FYHE and, therefore, essential when conceptualizing adjustment and integration.

Recently, several studies have again given voice to the first-year university student by qualitatively examining which student experiences they perceive to be at play during the transition period and, in doing so, identifying important facets and subfacets of the adaptation process. Trautwein and Bosse (2017), for instance, explored the first-year student experience by examining challenges (adjustments) that university students perceive as critical for a successful transition to HE. Their interviews with 25 students revealed 946 text segments that referred to critical incidents reported by the students. These narratives were categorized into 32 different critical student experiences, which the authors subsequently clustered into four dimensions: content-related, personal, organizational, and social requirements. Trautwein and Bosse (2017) suggested that the content-related, personal, and organizational dimensions were subdimensions of a higher-order academic integration dimension, which we will further describe here. The *content-related dimension* refers to experiences such as meeting curricular demand and pace, developing academic skills, and identifying performance and assessment standards. The *personal dimension* comprehends requirements such as scheduling learning activities, finding a mode of learning, or managing the workload. The *organizational dimension* encompasses, for instance, coping with the quality of teaching and learning, dealing with assessment conditions, or coping with formal regulations.

De Clercq et al. (2018) used semistructured interviews conducted in two steps with 17 freshmen from a university science department to unveil the constructs relevant to the student’s adjustment process and examine the dynamic interplay over time. The researchers identified four student themes important in the adjustment process (readiness, reaching personal goals, fighting an overwhelming program, and becoming a self-regulated learner), which they rooted in Nicholson’s (1990) Transition Cycle model. The first theme, *readiness*, concerns student experiences related to their perceived preparation regarding the necessary information, knowledge, and skills when entering HE. The *reaching personal goals* theme incorporates central constructs in the adjustment process that are essential for student’s success. It includes students’ feelings of competence (feeling confident in their abilities to succeed) and feelings of relatedness to their study (seeing value/relevance of or being passionate about the courses). The third theme includes student experiences regarding the heavy workload, the fast work pace, and the difficulty of course content and describes the central role of behavioral engagement in the adjustment process. Finally, the theme of *becoming a self-regulated learner* reflects the necessity of adopting a highly effective strategic management of their study program, which entails students being selective as to which courses to allocate their effort and adopting a flexible and selective way of learning. Furthermore, De Clercq et al. (2018) pointed out that complex relations exist between these uncovered constructs. For instance, they revealed that in this specific context and to succeed, a student both needs to carry out a certain quantity of work (behavioral engagement) in combination with good quality of that work (cognitive engagement).

These recent qualitative in-depth studies highlight the importance of various constructs in the first-year student academic experience, which can be related to several prevailing theories in transition literature. The results of the qualitative studies show, for instance, that first-year students report experiences related to their behavioral engagement (Fredricks et al., 2004; Christenson et al., 2012), self-regulation (e.g., Schunk and Zimmerman, 2012; Schunk and Greene, 2017), cognitive processing (Asikainen and Gijbels, 2017; Vermunt et al., 2017), and self-beliefs (e.g., Schunk, 1991; Bong and Skaalvik, 2003).

## The Present Study: The University College Students’ Perspective

The qualitative research summarized above was carried out in academic university contexts. A similar examination of students’ perceptions of the first-year academic experience in professional HE contexts, however, has not been conducted. Nevertheless, it is conceivable that university college students’ perceived realities of the academic transition are dissimilar from those of students in academic HE contexts. Firstly, in professional HE, the influx of students is more heterogenic than in academic HE. Indeed, students entering professional HE programs more often have technical or vocational prior education backgrounds and more often have encountered a study delay (Glorieux et al., 2014). Secondly, several lines of research have suggested that differences in learning environments might impact the freshmen’s academic adaptation process. For instance, it has been found that more activating learning environments result in higher levels of student engagement and learning (Umbach and Wawrzynski, 2005) and facilitate the development of good teacher–student relationships (Kember and Leung, 2005). Other research has demonstrated that the organization of the curriculum might also influence the first-year academic experience. In this light, Torenbeek et al. (2013), for example, found that a larger number of scheduled lectures in a study program led to lower class attendance and time spent on self-study. As detailed in the section “Introduction,” the learning environment in professional HE contexts differs from that in academic contexts. Both contexts have different aims and expectations of students and oftentimes differ in their institutional organization. Therefore, we believe an examination of the first-year student academic experience in professional HE programs is warranted.

In this study, we examine which academic experiences students in professional HE perceive to be the *most* important in the first semester of HE. We thus do not aim to make a clear-cut comparison of the academic transition between academic and professional HE contexts; rather, we give voice to the large group of first-year university college students by investigating their perceptions of the first-year student academic experience. Furthermore, we explicitly acknowledge the dynamic and temporal character of the transition process (e.g., Coertjens et al., 2017; Larose et al., 2019) by examining how these experiences occur over time. Hence, we have discerned the following research questions:

RQ1: What do first-year students in professional HE contexts perceive to be the most important experiences in their academic transition process during the first semester of their FYHE?

RQ2: How do the unveiled key academic experiences (RQ1) occur over different periods in the first semester of the FYHE?

## Materials and Methods

### Participants and Procedure

In this study, first-year students from 14 different study disciplines, such as nursing and social work, from a large (*N* > 12,000) Flemish university college participated. In order to pursue maximum variation in our qualitative data collection, we adopted a specific purposive sampling procedure (Cohen et al., 2011). In a first step, in November 2017, all students from the Dutch study disciplines in the HE institution (*N* = 3,225) completed questionnaires mapping out several aspects of their transition experience: self-efficacy, self-concept, emotion regulation, and social and academic adjustment. A considerable amount of literature has substantiated the relevance of these constructs in the prediction of important student outcomes, such as study success and well-being (e.g., Gratz and Roemer, 2004; Richardson et al., 2012; Buriæ et al., 2016). Based on the mean scores of the five variables, we explored whether we could identify distinctive student profiles using a latent profile analysis (Magidson and Vermunt, 2004). For this analysis, we only examined data from students who indicated that they had no previous experience with HE and who gave their informed consent (*N* = 2,168). As seen in Supplementary Appendix A, we were able to discern two moderate-sized groups [high (*n* = 727) and low (*n* = 346)] and one large middle group (*n* = 1,095). The low group represented students who reported particularly low scores regarding the five aspects of transition during their FYHE, while the high group reported high levels on these measures.

Respondents from these latter two distinct profiles (individuals of whom we could expect more outstanding transition experiences) who indicated that they wanted to participate in follow-up research (*n* = 939; high transition profile: *n* = 642; low transition profile: *n* = 297) were invited to take part in the qualitative data collection. Eventually, 104 students completed a reflective log at the start of the second semester of their FYHE (February 2018) in which they reflected on their experiences in the first semester. The sample consisted of 89 (86%) female and 15 (14%) male students. Furthermore, 81 (78%) respondents showed a high transition profile, while 23 (22%) students from the sample were classified as in the low transition profile group. The mean age of the respondents was 18.3 years.

### Data Collection

Inspired by the work of Trautwein and Bosse (2017), we adopted the fundamentals of the critical incident technique (CIT) for our data collection. The CIT, developed as an interview method (Chell, 2004), is recognized as an effective exploratory tool to collect self-reported experiences of essential real-life events (Butterfield et al., 2005). According to Flanagan (1954), a critical incident is a crucial event that makes a significant contribution—positively or negatively—to a certain phenomenon. In the present study, the basics of CIT were used to explore events that students perceived to be essential for their lived first-year academic experience during the first semester of their FYHE.

With the aim of eliciting critical incidents from a large number of students, we developed a paper-and-pencil reflective log (Supplementary Appendix B), which the respondents completed at the beginning of the second semester. In this log, we asked students to reflect upon the first semester and describe three experiences that they perceived for themselves to be critical regarding their academic adjustment to the new learning environment using open questions. These instances could be positive or negative but must have had an impact on the student. To map out experiences over a longer period—and in doing so acknowledging the process character of academic adjustment—a timeline was incorporated into the logs, which covered the complete first semester (September 2017 to January 2018). This timeline was divided into three time frames (Supplementary Appendix B), and students were requested to write down one critical incident for each period (thus, three critical incidents in total). According to Chell (2004), using such a timeline in CIT can be a “*visual aid serving several purposes*: *it focuses attention, enables the interviewee to relax, jogs the memory and enables the researcher to get a sense of the nature and chronology of any critical events*” (p. 48).

To ensure that students from all study disciplines would have the opportunity to participate in the data gathering, six time slots were organized at the institution at well-chosen time points in the first week of the second semester. Respondents watched a detailed 10-min instruction video before completing the log, which provided all participants with standardized instructions. Furthermore, the log was also accompanied by written instructions. Informed consent was obtained from all respondents and participating students had a chance to win a 100 EUR coupon.

### Analysis

NVivo software (v. 12.0 and 12.5) was used to code the data from the digitalized reflective logs. To classify the reported text segments into relevant themes (RQ1), a cross-case content analysis was undertaken (Krippendorff, 2004). In the first phase of this analysis, conforming to the CIT literature, we adopted an inductive open-coding approach to make a first abstraction of the critical incidents (Flanagan, 1954; Chell, 2004). For each of the reported critical incidents, the entire text segment was allocated to one or more pertaining codes, which resulted in a transparent overview of the data. This task was carried out by the first author. Subsequently, the emergent codes *and corresponding text segments* were thoroughly discussed with the second and third authors, and the need for redefinition and the development of additional (sub)categories in the coding scheme were identified. In this step, the concordance of our coding scheme with several prevailing theories in transition literature was extensively deliberated. However, the categorization of the reported experiences primarily relied on the commonalities between experiences we uncovered in the data and the focus the participants articulated in their text segments. In the second phase of the analysis, the first author modified the initial, tentative (sub)codes in the coding scheme, after which a second pass through the original data occurred. This second round of classification was again followed up by a discussion between the three authors. This iterative process of adaptation, inductive coding, and discussion was repeated two more times before the three authors reached a consensus on the allocation of text segments to codes.

To examine the trustworthiness of the resulting main themes, an independent coder recoded 15% of the reflective logs (16 of 104 logs; 48 of 312 narratives) in isolation, after which intercoder reliability was calculated using Cohen’s Kappa (Landis and Koch, 1977; Campbell et al., 2013). For all main themes, Cohen’s Kappa’s ranged from 0.866 to 0.998, indicating high to nearly perfect intercoder reliability (Landis and Koch, 1977). In a *post hoc* discussion, the independent coder had no suggestions for any additional adjustments to the coding scheme.

Finally, the cross-case approach was extended to investigate how the key academic experiences occurred over the three (see section “Data Collection”) discerned periods in the first semester (RQ2). Hereto, we created a frequency table at the level of the reported critical incidents, wherein references regarding key academic experiences were presented per theme per time frame.

## Results

### Themes of Critical Academic Experiences

The content analysis, which was based on the commonalities in the individual students’ narratives, resulted in the identification of nine main themes of experiences that students perceive to be critical in their academic transition process, and which can be placed under the umbrella terms academic adjustment and academic integration—as delineated in the theoretical framework. Regarding academic adjustment, or the active adaptation of behavior and attitudes as demanded by the HE environment, we unveiled five main themes of experiences: *(1) dealing with the organization of the study program*; *(2) organizing study work*; *(3) committing to the study*; *(4) following class and taking notes*; and *(5) processing learning content outside class.* The academic integration umbrella (i.e., the psychological state of being that results from an individual’s perception of fit with the new HE environment) comprehends four main themes of experiences: *(6) feeling competent*; *(7) feeling stressed*; *(8) feeling prepared*; and *(9) feeling supported*. In what follows, the nine main themes and their underlying subthemes are discussed (an overview of themes, subthemes, and the number of respondents referring to these pertaining themes is presented in Supplementary Appendix C).

#### Dealing With the Organization of the Study Program (*n* = 68)^1^

The first main theme under the academic adjustment umbrella includes experiences related to students coping with the way their HE study program is organized. It describes characteristics of the HE learning environment for which respondents do not hold themselves accountable, as they are inherently linked to the university college system with its specific conditions, planning, and expectations. These characteristics, however, provide situations and difficulties to which students must adapt. While some respondents provided a brief narrative of adapting to the characteristics below, others elaborated on how such characteristics affected their functioning or how they tried to overcome the difficulties (e.g., putting in extra effort or better self-regulating).

A first characteristic of the HE learning environment, on which 30 of the 104 students reported, was dealing with the mounting *quantity of work* in their new learning environment. The following student, for instance, described how she experienced a heavy workload and struggled to efficiently self-regulate (“*Organizing Study Work*,” see next paragraph):

*The group assignments and tasks piled up quickly! I had to take interviews, attend events, and do an internship. The pressure rises and you are continuously working for school. Soon I lost track of time and made misjudgments in my planning, but fortunately I made all my deadlines.* (45,F,H,T2)^3^

Furthermore, students needed to adjust to the *general planning* of the semester (*n* = 29). In this light, several students reported adjusting to the modular system (and its high pace), class schedules (e.g., long days, long free periods), numerous deadlines close to each other, lengthiness of the examination period, organization of the examination period (multiple exams planned in 1 day), the large number of practical exercises (which take away study time), and unexpected changes in the planning of the semester.

*In the beginning, I had difficulties with the long course days. Following classes until 7:30 pm or sometimes even 9:30 pm had a great influence on my daily routines, such as eating and sleeping. Consequently, fatigue was a big problem for me.* (82,F,L,T1)^4^

Regarding dealing with the organization of the study program, students also emphasized coping with the *expectations of teachers and the new system of evaluation* (*n* = 25). Indeed, several respondents wrote that it was hard to determine what was expected of them regarding the required level of knowledge and skills. Others explicitly reported that they did not experience any difficulties with this aspect of adjustment. Also, it was difficult for some students to foresee what questions on the examinations would look like and what was expected of them in more practical learning tasks. In other words, students had to make sense of expectations in the new learning environment.

*I found the first exams stressful. I had no clue of what the questions in higher education would be like since I didn’t get any intermediate tests in my study program*. (54,F,H,T3)^5^

Next to the aforementioned three facets, our analysis also uncovered two less significant themes of critical experiences related to the first main theme. Firstly, several students (*n* = 9) stated they experienced difficulties in *working with the online student platform*. Finally, some students were challenged by the *difficulty of certain learning tasks* (*n* = 6).

#### Organizing Study Work (*n* = 65)

According to the respondents, the first-year transition also demands adjustments regarding their self-regulation. This second main theme includes student experiences on dealing with executing three specific regulative tasks (i.e., planning, following planning, and evaluating the learning process), but also includes narratives that refer to cognitive self-regulation on a more abstract level. The regulative task mostly mentioned (*n* = 42) in the reflective logs was *making a planning*. In this subfacet, experiences are included wherein students explicitly report to (not) have made a planning. These narratives showed that many students experienced difficulties with this.

*The first weeks I had difficulties with planning assignments. A calendar with a weekly schedule didn’t work for me since we have lots of assignments and many of them are long term. I quickly lost sight of these assignments. Gradually I experienced the downside of my calendar. To keep an overview, I now work with a large whiteboard. This way, I continually see what needs to be done. It took me a few weeks to optimize this system.* (80,M,H,T1)

One such difficulty often reported by respondents (*n* = 17) was that they found it difficult to estimate how much time they would have to spend on a task, for instance, how much time it takes to process all the learning content for a specific exam. Other students (*n* = 14) reported that, although they had made a planning, this did not necessarily make them adhere to it. We, therefore, considered the subfacet *following the planning* as a second important regulative task.

*During the preparation period for the exams and the exam period itself, I experienced problems with planning. I found it hard to estimate what I was going to do when. I made a planning, but barely stuck to it.* (24,F,H,T3)

A third regulative task noted in the data was *evaluating the learning process*. Some students (*n* = 4) explicitly reported that, during the first semester, they reflected upon their achievement and their learning strategies (studying, planning) and altered their methods based upon these reflections.

*During this period, the exams of three courses [courses anonymized] started. The results weren’t great. I failed two of the three courses. Did I put too much time into my preparations? Did I memorize TOO hard and too detailed? I thought so, and I lost hope for a while… I needed to find a new study method.* (76,F,H,T2)

While the aforementioned subfacets comprise experiences on the level of specific regulative tasks, several respondents (*n* = 20) also referred to self-regulation on a more abstract level. The subfacet *organizing oneself* concerns whether students were able to organize their learning without explicitly referring to a specific regulative task. An illustration is provided by the following respondent who described difficulties in “organizing himself” and concerning “keeping an overview”:

*At the end of November, I again experienced many difficulties organizing myself and keeping an overview of my assignments and tests. I experienced an overflow of communication from the instructors regarding the exams, tests, and assignments that were approaching. I had difficulties processing it all.* (96,M,L,T2)

Students also detailed *dealing with subject matter efficiently* (*n* = 9), as they described how they made optimal use of their time to perform well and *making deadlines* (*n* = 6), which could be considered an outcome of (un)successfully organizing oneself.

Finally, students described experiences of *keeping up with their (study) work.* In total, 35 students reported (not) keeping up with their studies, detailing how they started on time with study tasks, daily repeated learning content, worked sufficiently during the semester, or rather procrastinated. It was clear from the data, however, that this latter category bears both an aspect of the students’ ability to self-regulate as well as an aspect of students committing to their studies (see the following section). Acknowledging this, we divided the narratives from these 35 students into the overarching themes *organizing study work* and *committing to the study* based on what respondents focused on in their quotes (self-regulation vs. behavioral engagement). In this light, narratives from a smaller group (*n* = 6) on “keeping up” were coded under “organizing study work.” For instance, the following quote shows that this student’s “inability to keep up with her work” principally centered around her inability to self-regulate her learning:

*In October and the beginning of November, I disregarded my theory because the exams still seemed so far away. On intermediate exams or tests, I didn’t score that well. Only later, I figured that in this period I didn’t have a good method to plan, keep up with my work, and prepare.* (83,F,L,T2)

#### Committing to the Study (*n* = 63)

The third main theme comprises experiences regarding students’ commitment to their studies. It generally represents students’ behavioral engagement (effort) and motivation regarding a variety of required tasks related to their study—or the lack thereof. The first subfacet of this third main theme was already mentioned in the main theme outlined above: *keeping up with the (study) work.* Interestingly, the majority of students who addressed keeping up in their narratives (29 from a total of 35) focused on the behavioral engagement facet rather than on the self-regulative facet, as illustrated by the following quote:

*I had very long school days, which made that, once at home, I didn’t feel like, and didn’t have the energy to keep up with my study work and rehearse the learning contents.* (93,F,H,T2)

This quote also further demonstrates the interrelatedness of the nine unveiled main themes of first-year student experiences. Indeed, it delineates how the organization of the study program (first main theme, i.e., longer school day) influenced this student’s commitment to her study.

A second facet of the commitment theme describes whether students are *motivated* to, for instance, study or go to class (*n* = 23). The quote of the following respondent exemplifies that she was generally motivated to start her HE career:

*I was very motivated to start. I knew I was going to have to work for it, but that didn’t hold me back to want to go in full for it.* (59,F,H,T1)

Another subfacet that emerged from students’ logs (*n* = 23) pertains to the extent to which students generally *make an effort to succeed or get good grades*. Of course, one could argue that students who keep up with their work (the first category of this main theme) also make an effort for their study. This latter category, however, comprises student reports that refer to effort on a more general level or in a specific period. Indeed, students who did not keep up with their work during the semester can still make an effort to succeed.

*During the preparation for my exams, I realized I was far behind in my studies. My achievements up till now weren’t great (however sufficient), and I didn’t master the learning content at all for the exams. The preparation period for my exams quickly became a series of days in the library, working from 8 am to 8 pm.* (79,F,L,T3)

In the context of committing to their study, students also reported on *whether they conscientiously went to* (*n* = 15) and/or *prepared for* (*n* = 11) *class*, as illustrated by the following quote of a motivated student who kept up her work and attended every class:

*In the beginning of the semester, I was very motivated. Several times a week, I went to the library to rehearse the learning content. I went to every class and made clear notes during the classes.* (4,F,H,T1)

#### Following Class and Taking Notes (*n* = 57)

Students *following class and taking notes* was a fourth major theme of critical experiences associated with academic adjustment. Several students (*n* = 15) described that they struggled with the *fast pace* of classes, wherein a lot of information is transferred, or specifically reported on adjusting to the *large class sizes* (*n* = 6). Other respondents (*n* = 12) wrote they experienced difficulties *concentrating* during classes (longer classes, new distractions).

*The lectures always take 3 h. For me, this was a big adjustment to concentrate for such a long time. At the beginning of the class, I was able to follow, but after about 50 min, I lost concentration. Especially because, all of a sudden, laptops and smartphones are allowed, I was distracted all of the time. After a short break, I was able to concentrate for a while, but this concentration was gone again after about 15 min.* (100,F,L,T1)

Interestingly, many students (*n* = 41) specifically described experiences related to *taking notes in class*. Most of these students seemed to struggle with several aspects of note-taking, such as not knowing what was important to note (*n* = 10) or not being able to keep up during note-taking (*n* = 14). Several students noticed afterward that the quality of their notes was not sufficient (*n* = 13). Finally, eight respondents described having switched from taking paper-and-pencil notes to taking digital notes on their laptop.

*At the beginning of the academic year, I had trouble with how to best make notes. For the course [course anonymized], in the first lessons I made paper and pencil notes, but this became one big chaos. After this, I switched to digital notes on my laptop, which enabled me to work more efficiently and clear.* (12,F,H,T1)

#### Processing Learning Content Outside Class (*n* = 43)

From the student perspective, studying the learning content in HE involved adjustment as well. Firstly, several students (*n* = 31) described experiences related to *processing the learning content on a more general level*. Most of these students reported that they struggled with studying or finding the right study method.

*For me, it generally was difficult to process the learning content of the lectures. I spent too much time summarizing the learning content, but I didn’t or barely got anything out of that.* (84,M,L,T1)

Next to the general difficulty with studying, students (*n* = 22) also specifically referred to particular *learning tasks* during their cognitive processing. In this light, students reported experiences related to making schemes, structuring/ordering the learning content, memorizing, and selecting important issues in the learning content.

*I found it difficult to select which issues were important and which were rather side issues. Because of this, my summaries were very wide-ranging.* (57,F,H,T1)

#### Feeling Competent (*n* = 39)

This first main theme incorporated under the integration umbrella incorporates experiences of students appraising their capabilities and performances. Several students (*n* = 30) referred to *feelings of confidence about whether they could handle their studies in general*. They described wondering they were doing well in their program or were in the right place. Such feelings were, for instance, repeatedly associated with how respondents dealt with failure or with feelings of lagging.

*For the course [course anonymized], we had to write a summary. Something that I never did before in high school. I worked hard on this assignment and hoped for the best, but it came out that the result wasn’t good at all. I was disappointed and had the feeling that I wasn’t good enough for university college.* (58,F,H,T2)

Other respondents (*n* = 12) described *more specific feelings of self-confidence related to successfully finishing particular courses or learning tasks*. For instance, the following respondent described that she feared being unable to process the information of an extensive study book:

*In the beginning, I was very afraid of the course [course anonymized] … They said that the PowerPoint slides weren’t sufficient. I didn’t know how to organize myself and select the most important elements out of the study book. The book counted 600 pages and I was really stressed out about that.* (19,F,H,T1)

#### Feeling Stressed (*n* = 28)

*Feelings of stress* also emerged from the above excerpt (19,F,H,T1) related to the respondent’s fear of not being able to process the information. This provides another illustration of the complex entanglement of themes of reported experiences. Indeed, we noticed that many reported occurrences of stress were strongly interrelated with how students felt they functioned as an HE learner. For instance, the following student further illustrated this association between stress and feeling competent by expressing that he had “no stress” while he had “a good feeling” concerning the upcoming exams, as he kept up with his study work:

*At the start of the exams, I had no stress and a good feeling because I had rehearsed the learning content of most courses earlier on. This led me to get good grades.* (28,M,H,T3)

#### Feeling Prepared (*n* = 20)

Several students described whether they felt prepared for their new learning environment in HE by their prior education. Firstly, students (*n* = 11) reported their secondary education provided them with the *necessary skills to function well in HE*. These skills enabled them to successfully make schemes, learn large amounts of material, make a planning, or follow the class and take notes:

*I received good preparation in high school. This helped me find a proper attitude during classes (take notes) and at home (summarizing, flipping the classroom).* (66,F,H,T1)

Secondly, students (*n* = 11) also described *whether they acquired important knowledge in secondary education*, as described by the following quote:

*On the first day of school when I met my peers, it became very clear to me that no one with my prior secondary education track would be in my class and that everyone was somewhat more intelligent than me. I noticed this especially in the first language classes where there were a lot of references to learning content from past years that I had not had.* (1,F,L,T1)

#### Feeling Supported (*n* = 14)

A final theme we discerned in the data related to students’ feeling supported when they experienced difficulties during their transition in the first semester of their FYHE. As stated in the student reports, this support might originate from staff (*n* = 10); externals, such as family (*n* = 3); and peers (*n* = 2).

*In the middle of November, I had found my feet. There were courses that were very difficult for me at first, but thanks to the help of the instructors, everything went well. During the first semester, I received a lot of help from my teachers, who guided me well.* (27,M,H,T2)

### Occurrence of Key Academic Experiences Over Time

In accordance with RQ2, we considered how the references regarding different main themes of experiences were distributed across the three periods distinguished in the reflective logs by creating a frequency table at the level of the reported critical incidents (Table 2).

**TABLE 2:** References per theme per time frame.

**Theme**	**Period 1**	**Period 2**	**Period 3**	***Total***
***Academic adjustment***				
*Dealing with organization of the study program*	30	37	33	*100*
*Organizing study work*	20	31	35	*86*
*Committing to the study*	27	30	34	*91*
*Following class and taking notes*	48	11	4	*63*
*Processing learning content outside class*	13	17	27	*57*
***Academic integration***				
*Feelings of competence*	11	20	25	*56*
*Feelings of stress*	5	10	16	*31*
*Feeling prepared*	10	8	5	*23*
*Feeling supported*	8	5	3	*16*

*Total*	*172*	*169*	*182*	*523*

Table 2 shows that, for most main themes, references were not evenly distributed across the three periods. However, themes were also not exclusively related to a certain period. Firstly, experiences concerning “following class and taking notes,” “feeling prepared,” and “feeling supported” were mostly reported upon at the beginning of the first semester (period 1: September to October) and showed a decline in reporting toward the end of the semester (period 2: end of October to the beginning of December; and subsequently period 3: the beginning of December to January). Secondly, the opposite trend can be observed for the themes “processing learning content outside class,” “feelings of competence,” and “feelings of stress,” which seem especially critical at the end of the first semester when students prepare for and take their exams. Finally, a constant trend was apparent for the remaining three themes: “dealing with the organization of the study program,” “organizing study work,” and “committing to the study,” as experiences were more evenly distributed. In sum, these observed trends in our data provided first indication that the importance of the themes seems to shift during the first semester, but for some themes more than for others.

## Discussion and Conclusion

Given that an understanding of how first-year students in professionally oriented HE programs experience their academic transition process is lacking, the present study set out to give voice on this matter from a large group of students. Our results, based on 104 reflective logs that were completed by purposively selected students from 14 different study disciplines, reflect two main findings.

### Nine Main Themes of Critical Academic Experiences

Firstly, we unveiled nine main themes of academic experiences that university college students perceived to be critical and which can be regarded as central constructs that are at play in the multifaceted academic transition process in professional HE contexts: (1) dealing with the organization of the study program, (2) organizing study work, (3) committing to the study, (4) following class and taking notes, (5) processing learning content outside class, (6) feeling competent, (7) feeling stressed, (8) feeling prepared, and (9) feeling supported. Although for the formulation of the main and subthemes we inductively adhered to the contents respondents focused on in their logs, several of these themes resonate with existing concepts in the transition literature, such as self-regulation (e.g., Schunk and Zimmerman, 2012; Schunk and Greene, 2017), behavioral and motivational engagement (Pekrun and Linnenbrink-Garcia, 2012), cognitive processing (Asikainen and Gijbels, 2017; Vermunt et al., 2017), self-beliefs (self-concept and self-efficacy; e.g., Schunk, 1991; Bong and Skaalvik, 2003), stress (Robotham and Julian, 2006; Friedlander et al., 2007), feeling prepared by secondary education (Torenbeek et al., 2010; Noyens et al., 2020), or feeling supported (Tao et al., 2000). Our study thus corroborates the importance of this specific set of constructs in the first-year academic experience in professional HE contexts and provides rich descriptions of how university college students perceive these concepts.

On a higher level, the academic experiences incorporated in the nine main themes could be categorized as two types of experiences. Firstly, the first five themes cover experiences regarding the active adaptation of behavior and attitudes as demanded by the HE environment (Baker and Siryk, 1984; Larose et al., 2019), which—according to our broad operational definitions outlined in the theoretical framework—fall under the adjustment umbrella. Secondly, the last four themes refer to a psychological state of being that results from an individual’s perception of fit with the new HE environment (Hausmann et al., 2007; Wolf-Wendel et al., 2009; Strayhorn, 2012), thus belonging under the integration umbrella.

Interestingly, most of the main and subthemes we described were also mentioned in the qualitative studies by Trautwein and Bosse (2017) and/or De Clercq et al. (2018), which described the academic transition in academic contexts. Although it is difficult to make an exact comparison of the significance of our themes relative to the academic HE context due to the differences in study designs and reporting, some noticeable similarities and differences with the findings of these studies merit highlighting. For instance, in both academic and professional contexts, many students experience difficulties adjusting to a heavier workload. Trautwein and Bosse (2017), however, also emphasized the fact that many students were challenged by the difficulty of their subjects, while in our study, only a few university college students reported struggling with this. Another striking aspect of our findings in comparison with research in academic contexts concerns the high number of students reporting on following class and taking notes. Although this was touched upon by respondents in Trautwein and Bosse’s (2017) study, our results suggest that this theme is more important for university college students.

### Importance of the Main Themes Seems to Shift Over Time

A second main finding of this study is that reports on the nine uncovered main themes are not evenly distributed over the three discerned periods in the reflective logs although every theme is mentioned in every period. More precisely, three overall trends were discerned in the data: (1) a declining trend in reporting toward the end of the semester for “following class and taking notes,” “feeling prepared,” and “feeling supported”; (2) an upward trend in reporting toward the end of the semester for “processing learning content outside class,” “feelings of competence,” and “feelings of stress”; and (3) a constant trend in reporting across the three discerned periods for “dealing with the organization of the study program,” “organizing study work,” and “committing to the study.” These results highlight the importance of considering the dynamic character of academic transition (e.g., Roland et al., 2016; Coertjens et al., 2017; De Clercq et al., 2018). We would like to emphasize that our qualitative data do not allow for inference to a larger population, as the sample is too limited. The observed trends should, therefore, be interpreted with caution. Nevertheless, our results do provide first indications that although the different themes of academic experiences, in general, seem to be more important at different times in the first semester, they all appear to be meaningful throughout the whole semester.

### Reflecting on a Complex Entanglement Between Constructs

An interesting reflection we made during the analysis is that the different themes and subthemes of academic experiences (i.e., constructs at play during the academic transition to professional HE) are strongly interrelated. In the “Results*”* section, three such relationships were highlighted: (1) how the high quantity of work in the study program was associated with a student’s ability to effectively self-regulate her study work (45,F,H,T2; p. 10); (2) how one student’s commitment toward his study was negatively influenced by the long, exhausting school days (93,F,H,T2; p. 12); and (3) how a student’s self-perceptions might influence feelings of stress (19,F,H,T1; p. 14). Several such detailed examples of how university college students encounter specific and complex entanglements of themes of academic experiences could be provided here. This observed entanglement emphasizes that our classification of first-year academic experiences should not mask the fact that students encounter a complex web of challenges and experiences during their transition to HE (Trautwein and Bosse, 2017; De Clercq et al., 2018), and further corroborates the statement of Tinto (2012) that transition is “*a very complex, quite fluid situation that need not be experienced in the same fashion by every student*” (p. 94). However, although our research design proved to be valuable for its main purpose (identifying key constructs in first-year students’ academic transition), we found it to be less suited to further explore the entanglement of constructs, as we did not specifically ask students to reflect hereupon; consequently, students did not report on this consistently. Indeed, examining the relationships between each pair of uncovered themes (and subthemes) merits its own research question and was beyond the scope of the present study.

### Future Perspectives and Limitations

Our study brings to the fore nine constructs central in the academic transition process in professional HE as perceived by university college students. However, the observed entanglement of themes, as mentioned above, corroborates the conception of the transition process as an aggregation of specific microprocesses—revolving around the nine central constructs—that differ between individuals in their respective situations (De Clercq et al., 2018). This reflection challenges the idea that variable-centered quantitative study designs can accurately capture the specific student experiences occurring during the academic transition and thus, advocate for the use of in-depth qualitative research methods when further grasping the first-year students’ academic experience. Nevertheless, for quantitative future research that aims to explore trends on a larger scale in professional higher education, we endorse the statement of De Clercq et al. (2018) that the student academic adaptation process *“cannot be seen as the sum of adaptive factors but should rather be seen as a complex recipe where each ingredient needs to be taken into account and accurately measured*” (p. 84).

In this light, our review of the literature demonstrated that scholars adopt a variety of subfacets when interpreting the academic adjustment and integration constructs and that these terms are often used interchangeably (see Table 1 for illustrations). Moreover, oftentimes, it is not explicitly clarified on which theoretical basis the choice of a specific set of subfacets rests. Firstly, our study provides theoretical clarity on what we understand under integration and adjustment (see section ‘Academic Adjustment and Integration: Two Complementary Perspectives’). Secondly, it provides a foundation for scholars when conceptualizing and operationalizing the first-year student academic transition in terms of (1) the active adaptation of behavior and attitudes as demanded by the HE environment and (2) the psychological state of being that results from an individual’s perception of fit with the new HE. We argue that such interpretations (covered subfacets) should, at least, incorporate the respective constructs that were unveiled in the present study, as they are perceived to be critical by first-year university college students.

Another perspective for future research builds on the results of this study that provide first indications that university college students’ perceived realities of the academic transition might be different compared with those of students in academic settings. Indeed, although the same constructs are at play in both contexts, the perceived importance of these concepts seems to differ—as was mentioned above. Future research should further focus on contrasting both HE contexts by using designs specifically developed for this purpose. Such a design might, for instance, take the form of the simultaneous deployment of the reflective logs used in the present study in large samples of students in both academic and professional HE contexts and subsequent comparison of the data between these two groups.

When interpreting the findings of the present study, some limitations need to be considered. Firstly, the data used in the present study was self-reported and retrospective. Respondents thus needed to rely on their memory to reconstruct earlier processes, which might lead to a certain level of bias in the results (e.g., Nisbett and Wilson, 1977; Pekrun, 2020). Future research that adopts an online approach, where students are longitudinally tracked throughout the first semester, might provide more valid and reliable data and conclusions. It should, however, be mentioned here that an important advantage of using the critical incidents technique is that, even if collected accounts are retrospective, respondents usually have a good recall of these events since they are “critical” (Chell, 2004).

This study is also limited by the fact that it only considers academic experiences in the first semester of FYHE, as it is acknowledged that the first-year students’ academic transition process is not finished after the first semester (e.g., Gale and Parker, 2014; Coertjens et al., 2017). Thus, although many scholars agree that first-year students are confronted with academic challenges especially in the first semester of FYHE (e.g., Clinciu, 2013; Martens and Metzger, 2017; Bowman et al., 2019), it seems worthwhile to also consider students’ academic experiences in professional contexts during the second semester and into subsequent years. Finally, it should be pointed out that students from the low transition profile and male students are underrepresented in the present study. Indeed, in the HE institution’s first-year student population, 32% of the students were classified as in the low transition profile group and 35% were male, while these proportions in our sample were—as mentioned in the section “Materials and Methods”—only 22 and 14%, respectively.

Despite these limitations, the present study provides administrators in professional HE contexts with some clear handles and guidelines for the development of guidance and support initiatives. We recommend that the design of such initiatives should minimally comprise the nine main themes of academic experiences described in this study, which are perceived to be critical by first-year university college students. Furthermore, HE administrators should also consider the temporal aspect of the academic transition. More precisely, they should determine which specific first-year students’ challenges should be focused on at which moment in the first semester. In this light, our results indicate that a guidance trajectory should ideally start by focusing *especially* on students’ challenges regarding “following class and taking notes,” “feeling prepared,” and “feeling supported,” while the themes “processing learning content outside class,” “feelings of competence,” and “feelings of stress” might be covered somewhat later in the semester. “Dealing with the organization of the study program,” “organizing study work,” and “committing to the study,” on the other hand, should be nourished throughout the whole semester.

## Data Availability Statement

The raw data supporting the conclusions of this article will be made available by the authors, without undue reservation.

## Ethics Statement

The studies involving human participants were reviewed and approved by Ethics Committee for the Social Sciences and Humanities (EA SHW), University of Antwerp. The patients/participants provided their written informed consent to participate in this study.

## Author Contributions

JW conducted the study. LC and VD contributed to the design of the study, discussed the coding, and participated in the writing of the manuscript. All the authors contributed to the article and approved the submitted version.

## Conflict of Interest

The authors declare that the research was conducted in the absence of any commercial or financial relationships that could be construed as a potential conflict of interest.

## Footnotes

1 This theme was reported by 68 of the 104 respondents.

3 45,F,H,T2 = respondent 45, female, high social and academic integration profile, period 2 (end of October to beginning of December)

4 T1 = September to October

5 T3 = beginning of December to January
