# Supplementary material for: Entering Higher Professional Education: Unveiling First-Year Students’ Key Academic Experiences and Their Occurrence Over Time
Source: Front Psychol. 2021 Feb 24;12:577388. doi: 10.3389/fpsyg.2021.577388 (PMC7943626; doi:10.3389/fpsyg.2021.577388)
Supplement: Supplementary file 1 [file Data_Sheet_1.docx]

# Appendices

***Appendix A***

For the selection of our participants for the qualitative data gathering, we adopted a purposive sampling procedure (see *Procedure and Participants*). Hereto, we carried out a latent profile analysis (latent variable mixture analysis with continuous cluster indicators), using Mplus 7.4 (Muthén and Muthén, 2012). This profile analysis was conducted based upon the means of five validated quantitative measures that represent important aspects of freshmen’s FYHE student experience (e.g., Richardson et al., 2012): *academic self-efficacy* (Item example: “I think that I am good at studying”; *α* = 0.83), *academic self-concept* (“I think I can handle this study program”; *α* = 0.80), *emotion* *regulation* (“When I’m upset, I have difficulty getting work done”; *α* = 0.87), *social* *adjustment* (“I am meeting as many people, and making as many friends as I would like at my university college”; *α* = 0.82), and *academic adjustment* (“I have been keeping up to date on my academic work”; *α* = 0.84). All scales were based on previously validated instruments (Donche and Van Petegem, 2008; Van Soom and Donche, 2014; Beyers and Goossens, 2002; Gratz and Roemer, 2004; Wouters et al., 2011) and had good internal consistency and construct validity.

In accordance with the study of Fryer (2017), several fit indices were employed in estimating the optimal number of profiles arising from the mixture models. Firstly, lower values of Akaike’s information criterion or AIC (Akaike, 1987), Bayesian information criterion (BIC; Schwartz 1978), and sample-size adjusted BIC (Sclove, 1987) indicate better model fit. Secondly, the Lo–Mendell–Rubin likelihood ratio test (Lo et al., 2001) and the Vuong–Lo–Mendell–Rubin likelihood ratio test (Vuong, 1989) provide a test of whether a model significantly improves after the addition of one more profile. According to Asparouhov and Muthén (2012), in these latter two tests, a nonsignificant *p* value then rejects the *k* − 1 profile model in favor of the *k* profile model. Lastly, we also considered an entropy criterion in deciding on the best solution for the number of profiles. An entropy value closer to 1 represents a better classification of a sample into subgroups (see Celeux and Soromenho, 1996 for more information). The fit indices and entropy levels for the latent profile analyses are presented in **Table 3**.

In addition to the aforementioned fit indices and entropy levels, the relative size of the classes and their theoretical meaningfulness were also taken into account when determining the resulting number of profiles. Finally, after the best solution had been determined, ANOVAs were carried out to evaluate the variance explained by the resulting profiles for each variable (Fryer, 2017).

**TABLE 3** | Overview of fit indices for latent profile analyses.

| **Profile** | **BIC** | **∆BIC** | **BIC adjusted** | **∆BIC adjusted** | **AIC** | **∆AIC** | **Entropy** | **Vuong–Lo**  **–Mendell–Rubin** | **Lo–Mendell–Rubin** |
| --- | --- | --- | --- | --- | --- | --- | --- | --- | --- |
| 1 profile | 23,947.14 |  | 23,915.37 |  | 23,890.33 |  |  |  |  |
| 2 profiles | 22,243.14 | 1,704.00 | 22,192.31 | 1,723.06 | 22,152.24 | 1,738.09 | 0.73 | <0.001 | <0.001 |
| 3 profiles | 21,838.20 | 404.94 | 21,768.30 | 424.00 | 21,713.21 | 439.03 | 0.71 | <0.001 | <0.001 |
| 4 profiles | 21,765.02 | 73.18 | 21,676.06 | 92.25 | 21,605.93 | 107.27 | 0.68 | 0.18 | 0.18 |
| 5 profiles | 21,655.72 | 109.30 | 21,547.70 | 128.36 | 21,462.55 | 143.38 | 0.75 | 0.02 | 0.02 |
| 6 profiles | 21,591.47 | 64.26 | 21,464.38 | 83.32 | 21,364.20 | 98.35 | 0.72 | 0.15 | 0.15 |

Following the procedure adopted by Fryer (2017) and Nylund-Gibson et al. (2014), in the first phase, we inspected **Table 3** for the last relatively large decrease in the BIC value (“the elbow”), as a guide in selecting the best fitting model among those considered. This guide suggests a three-group solution, which is further substantiated by the Lo–Mendell–Rubin test and the Vuong–Lo–Mendell–Rubin test; these tests both indicate that adding a fourth group to the model does not significantly improve the model. Moreover, in the four-profile solution, the entropy level drops to 0.68. Applying the “elbow” principle to the AIC and adjusted BIC, then, further suggests to favor the three-group model.

Furthermore, the three-profile solution is theoretically sound. It exists of one large “middle” group (*N*= 1,095) and two moderate-sized groups: “high” (*N*= 727) and “low” (*N*= 346). The low group represented students who reported particularly low experiences regarding the abovementioned aspects of the FYHE student experience, while the high group reported high levels on these measures. The middle group, then, is located in between those two profiles. ANOVAs and subsequent *post hoc* analyses show that all three profiles significantly differ from each other regarding the modeled variables (**Table 4**). Finally, **Table 4** also shows that the three latent groups explain an acceptable to substantial amount of variance (*R*²) in each of the five indicators.

|  | Low group mean | Mid group mean | High group mean | *p* | *F* | *R*² |
| --- | --- | --- | --- | --- | --- | --- |
| Self-efficacy | 1.97 | 3.00 | 3.76 | <0.001 | 2,241.69 | 0.672 |
| Self-concept | 2.62 | 3.23 | 3.85 | <0.001 | 1,181.32 | 0.519 |
| Emotion regulation | 2.77 | 3.19 | 3.60 | <0.001 | 110.82 | 0.093 |
| Social adjustment | 3.42 | 3.81 | 4.12 | <0.001 | 126.84 | 0.104 |
| Academic adjustment | 2.38 | 3.02 | 3.81 | <0.001 | 1,017.26 | 0.486 |

**TABLE 4** | ANOVA results and explained variance.

*Note:* *Post hoc* analyses show that all within-row means are significantly different from each other.

*References*

Akaike, H. (1987). Factor analysis and AIC. *Psychometrika, 52*, 317-332.

Asparouhov, T., and Muthén, B. (2012). Using Mplus TECH11 and TECH14 to test the number of latent classes. *Mplus Web Notes: No. 14*. [http://statmodel.com/examples/webnotes/webnote14.pdf. Accessed 2 July 2019](http://statmodel.com/examples/webnotes/webnote14.pdf.%20Accessed%202%20July%202019).

Beyers, W., & Goossens, L. (2002). Concurrent and predictive validity of the Student Adaptation to College Questionnaire in a sample of European freshmen students. *Educational and Psychological* *Measurement, 62,* 527‐538.

Celeux, G., and Soromenho, G. (1996). An entropy criterion for assessing the number of clusters in a mixture model. *Journal of classification, 13*(2), 195-212.

Donche, V., & Van Petegem, P. (2008). The validity and reliability of the short inventory of learning patterns. In E. Cools, H. Van den Broeck, C. Evans, & T. Redmond (Eds.), *Style and cultural differences: how can organisations, regions and countries take advantage of style differences* (pp. 49–59). Ghent: Vlerick Leuven Gent Management School.

Fryer, L. K. (2017). (Latent) transitions to learning at university: A latent profile transition analysis of first-year Japanese students. *Higher Education*, *73*(3), 519-537.

Gratz, K. L., & Roemer, L. (2004). Multidimensional assessment of emotion regulation and dysregulation: Development, factor structure, and initial validation of the difficulties in emotion regulation scale. *Journal of psychopathology and behavioral assessment*, *26*(1), 41-54.

Lo, Y., Mendell, N. R., and Rubin, D. B. (2001). Testing the number of components in a normal mixture. *Biometrika*, *88*(3), 767-778.

Muthén, L. K., and Muthén, B. O. (2012). *Mplus User’s Guide. Seventh Edition*. Los Angeles, CA: Muthén and Muthén.

Nylund-Gibson, K., Grimm, R., Quirk, M., and Furlong, M. (2014). A latent transition mixture model using the three-step specification. *Structural Equation* *Modelling: A Multidisciplinary Journal*, *21*(3), 439–454.

Schwarz, G. (1978). Estimating the dimension of a model. *The annals of statistics*, *6*(2), 461-464.

Sclove, S. L. (1987). Application of model-selection criteria to some problems in multivariate analysis. *Psychometrika*, *52*(3), 333-343.

Van Soom, C., & Donche, V. (2014). Profiling first-year students in STEM programs based on autonomous motivation and academic self-concept and relationship with academic achievement. *PLoS One, 9*(11), e112489.

Vuong, Q. H. (1989). Likelihood ratio tests for model selection and non-nested hypotheses. *Econometrica*: *Journal of the Econometric Society*, *57*(2) 307-333.

Wouters, S., Germeijs, V., Colpin, H., & Verschueren, K. (2011). Academic self‐concept in high school: Predictors and effects on adjustment in higher education. *Scandinavian journal of psychology*, *52*(6), 586-594.

***Appendix B***


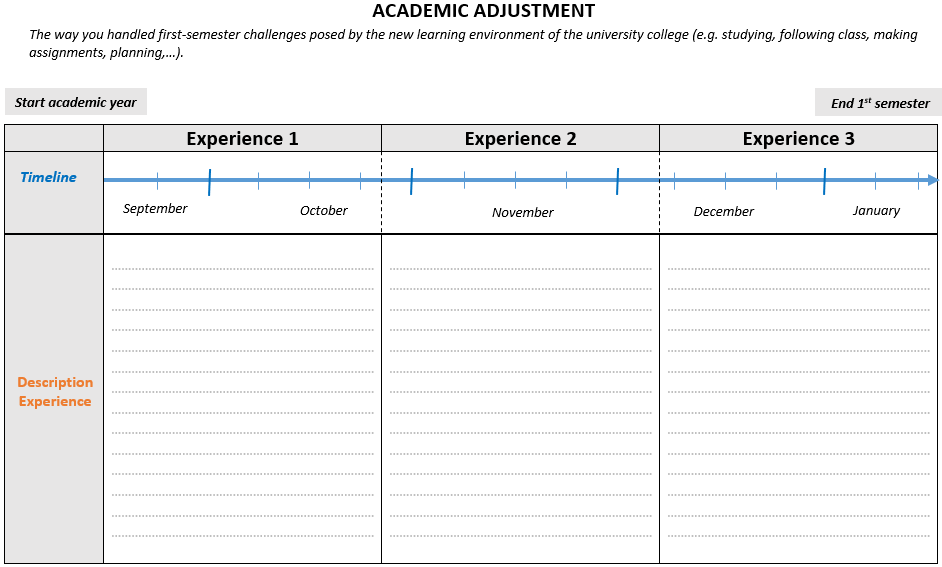


***Appendix C***

**TABLE 5** | Themes of academic experiences, depicted in descending order of number of respondents referring to the pertaining themes.

| **(Sub)theme** | **Number of respondents referring to theme** |
| --- | --- |
| **Academic adjustment** |  |
| 1. *Dealing with organization of the study program* | *68* |
| Quantity of work | 30 |
| General planning | 29 |
| Expectations of teachers and the new system of evaluation | 25 |
| Working with the online student platform | 9 |
| Difficulty of certain learning tasks | 6 |
| 1. *Organizing study work* | *65* |
| Making a planning | 42 |
| Organizing oneself | 20 |
| Following the planning | 14 |
| Dealing with subject matter efficiently | 9 |
| Making deadlines | 6 |
| Keeping up with the (study) work | 6 |
| Evaluating the learning process | 4 |
| 1. *Committing to the study* | *63* |
| Keeping up with the (study) work | 29 |
| Being motivated | 23 |
| Make an effort to succeed or get good grades | 23 |
| Conscientiously going to class | 15 |
| Conscientiously preparing for class | 11 |
| 1. *Following class and taking notes* | *57* |
| Taking notes in class (e.g., not knowing what is important to note) | 41 |
| Struggle with fast pace of classes | 15 |
| Difficulties to concentrate | 12 |
| Adjusting to large class size | 6 |
| 1. *Processing learning content outside class* | *43* |
| Processing the learning content on a more general level | 31 |
| Particular learning tasks | 22 |
| **Academic integration** |  |
| 1. *Feeling competent* | *39* |
| Feeling confident in generally handling the study | 30 |
| Feeling confident in successfully finishing particular courses or learning tasks | 12 |
| 1. *Feeling stressed* | *28* |
| 1. *Feeling prepared* | *20* |
| Necessary skills to function well in HE | 11 |
| Acquired important knowledge in secondary education | 11 |
| 1. *Feeling supported* | *14* |
| Staff | 10 |
| Family | 3 |
| Peers | 2 |
